# Statistics of the Mortality Following Ligature of the Femoral Artery

**Published:** 1849-12

**Authors:** 


*Statistics of the Mortality following Ligature of the Femoral Artery.* By GEORGE NORRIS, M. D. (Am. Jour. Med. Science, Oct., '49).

*Mortality.*—Of two hundred and four cases in which the femoral artery has been ligatured by the Hunterian method, one hundred and fifty-four recovered and fifty died. Six of the patients who recovered, undergoing amputation in consequence of gangrene of the limbs.

*Disease or Injury.*—The artery was tied in one hundred and fifty-five, for the cure of popliteal aneurisms; in twenty-two for femoral aneurisms; in six, for aneurismal tumors of the leg; in four, for pulsating tumors of the head of the tibia or condyle of the femur; in five, for varicose aneurisms; in three, for rupture of the artery, accompanying fractures of the leg or thigh; in eight, in order either to prevent or abate inflammation after wounds and dislocations, or to arrest hemorrhage, either primary or secondary; and in one, for a supposed popliteal aneurism.

Of the one hundred and eighty-eight cases in which the operation was done for the cure of aneurisms, one hundred and forty-two were cured, and forty-six died.

*Sex.*—Of one hundred and eighty-three cases in which this is noted, one hundred and seventy-seven occurred in males, and six in females.

*Right or left Side.*—Of one hundred and twenty-four cases of aneurisms where the side is mentioned, sixty-three were on the right, and sixty-one on the left side.

*Age.*—The age is noted in one hundred and sixty-four cases of aneurisms, and of these there were

| | | | |
|---|---|---|---|
| Under 10 years, | 1 | a true aneurism. | |
| Between 10 and 20 years, | 4 | " | " |
| " 20 and 30 " | 30 | " | " |
| " 30 and 40 " | 72 | " | " |
| " 40 and 50 " | 40 | " | " |
| " 50 and 60 " | 14 | " | " |
| 60, and above it, | 3 | " | " |
| Total, | 164 | " | " |

*Period the Ligature separated.*—In one hundred and thirty-seven cases in which the ligature separated spontaneously, it came away in ninety-one before the twentieth day; in thirty-seven, between the twentieth and thirtieth days; in fourteen, between the thirtieth and fortieth days; and in five, beyond the fortieth day. The longest period to which it remained, was the sixtieth day, and the shortest the fifth day.

---

*Return of Pulsation* in the aneurismal tumor, after the application of the ligature, was observed in nineteen cases.

*Hemorrhage* after the operation occurred in twenty-four cases, of which twelve died and twelve were cured, one of the latter being with the loss of the limb.

*Suppuration* of the sac occurred after the operation, in sixteen cases; and of these, six died and ten did well.

*Gangrene* of the limb followed in thirty-one out of the two hundred and four cases contained in the tables, and it in no case occurred, except where the operation was done for aneurisms.

*Cause of Death.*—Of the two hundred and four cases contained in the tables, fifty died. Of these twenty-three died from mortification of the limb; eight from hemorrhage; five from phlebitis; three from tetanus; two from hectic and diarrhœa; one from thoracic inflammation and abscess in the course of the artery; one from sloughing of the sac; one from the bursting of an aneurism of the aorta within the pericardium, twelve weeks after the operation; one from fever; one from absorption of pus; and in four the cause is not noted.

*Mistakes in Diagnosis.*—In four of the cases included in the tables mistakes in diagnosis occurred. In the first, the precise nature of the tumor could not be ascertained, and it was punctured before the operation. In the second, the tumor was mistaken for an abscess, and opened a week previous to the operation; after bleeding three pints, it ceased spontaneously. In the third, the tumor was the size of a goose's egg, distinctly pulsated, and was said to have shown itself suddenly, while walking, three weeks previously. Compression was at first attempted, and afterwards the artery was tied—notwithstanding which, the swelling continued to increase. On post-mortem examination, it was found to consist partly of a fibrous tissue divided into lobes, and in part of a soft substance containing cells filled with a serous fluid. The artery ran over the tumor between two of the sacs, and through these its pulsations had been so communicated as to give the sensation of the whole tumor pulsating. The right lung was converted into a brain-like mass. In the fourth, the patient was a black woman, in whom there existed great swelling of the whole limb, but particularly of the popliteal region. Suspecting deep suppuration, an incision, an inch in length, was made into it, from which nothing but a little serum, slightly colored with blood, escaped. The integuments in the popliteal region afterwards ulcerated, and some days subsequently there was a profuse hemorrhage from the part when the artery was tied.
—*Charleston Med. Jour. and Review.*

